# T cell receptor-engineered T cells for leukemia immunotherapy

**DOI:** 10.1186/s12935-018-0720-y

**Published:** 2019-01-03

**Authors:** Yikai Zhang, Yangqiu Li

**Affiliations:** 1 Key Laboratory for Regenerative Medicine of Ministry of Education, Institute of Hematology, School of Medicine, Jinan University, 601 Huang Pu Da Dao Xi, Guangzhou, 510632 People’s Republic of China; 2 Department of Hematology, First Affiliated Hospital, Jinan University, Guangzhou, 510632 China

**Keywords:** T cell receptor-engineered T cells, Immunotherapy, Leukemia

## Abstract

At present, refractory and relapse are major issues for leukemia therapy and a major cause of allogeneic hematopoietic stem cell transplant failure. Over the last decade, many studies have demonstrated that adoptive cancer antigen-specific T cell therapy is an effective option for leukemia therapy. Recently, T cell immunotherapy studies have mainly focused on chimeric antigen receptor- and T cell receptor-engineered T cells. Clinical trials involving chimeric antigen receptor-engineered T cells have been a major breakthrough and became a novel therapy for leukemia. As another potential therapy for leukemia, clinical application of TCR-engineered T cells remains in its infancy. This article presents a review of the current status of anti-leukemia immunotherapy using leukemia antigen-specific TCR-engineered T cells.

## Background

Refractory and relapse are major issues for leukemia therapy and a major cause of allogeneic hematopoietic stem cell transplant (HSCT) failure. Adoptive cytotoxic T lymphocyte (CTL) infusion has been demonstrated to be effective for treatment of relapse chronic myelogenous leukemia (CML) after HSCT, cytomegalovirus (CMV)—mediated disease, and Epstein–Barr virus (EBV)—positive B cell lymphomas or post-transplantation lymphoproliferative disorders (PTLPDs) in clinical trials [1, 2]. In particular, adoptive tumor and leukemia antigen-specific T cell therapy are the most effective options. However, it is difficult to generate sufficient numbers of antigen-specific CTLs for the treatment of each patient in vitro in a short period of time, particularly for acute leukemia patients, whose blood include large numbers of leukemia blast cells, and the percentage of T cells is relative low, limiting the application of this approach. Moreover, T cells from cancer and leukemia patients have an exhausted phenotype and low activation, which also limits their application [3]. For example, telomerase (TERT)-specific CTLs, which were identified in blood in B-cell chronic lymphatic leukemia (B-CLL) patients, display low functional avidity [4].

**Fig. 1:**
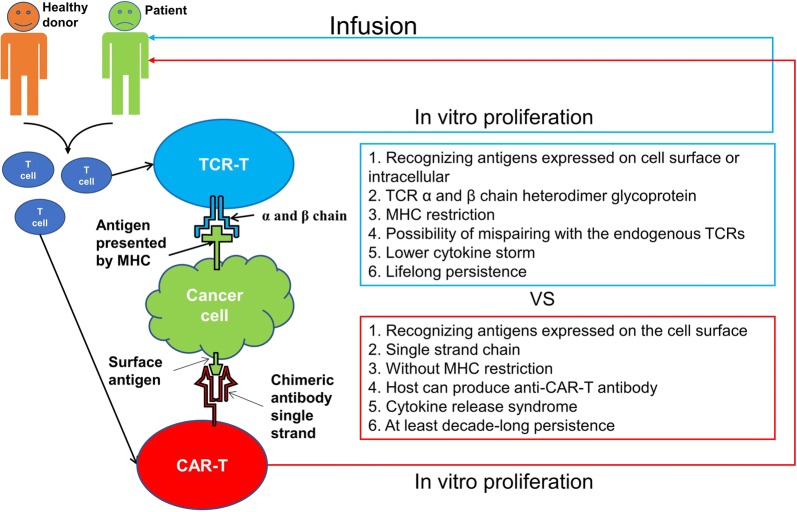
Characteristics of TCR- and CAR-engineered T cells

Currently, T cell immunotherapy has focused on chimeric antigen receptor (CAR)- and T cell receptor (TCR)-engineered T cells in which T cells have been engineered to express artificial receptors targeting leukemia or other tumor cells. This approach has emerged from principles of basic immunology to paradigm-shifting clinical immunotherapy. Clinical trials with cluster of differentiation 19 (CD19)-specific CAR-T cells have demonstrated durable remission in adult and children patients with advanced B cell leukemia and lymphomas [5, 6]. In general, the adoptive immune responses mediated by antigen-specific CTLs are decisively performed by TCRs. The technique of using TCR-modified T cells to provide wider opportunities to redirect T cells against viruses or tumor antigen-bearing cells was reported as early as 1996. Brocker et al. showed that a chimeric TCRβ chain consisting of a single-chain Fv portion derived from a monoclonal antibody and the full TCRβ chain is capable of functionally assembling with endogenous TCR/CD3 components and transferring antibody specificity and TCR specificity into TCRβ^−^ or TCRβ^+^ T cells [7]. Then, the genes encoding TCRα and β in a melanoma-associated antigen peptide-1 (MART-1)-specific, human lymphocyte antigen (HLA)-A2-restricted human T cell clone was efficiently transferred and expressed in human peripheral blood T cells of patients with melanoma. These TCR-modified T cells displayed specific anti-tumor reactivity in vitro and could potentially offer treatment for patients with metastatic melanoma [8]. Unlike the rapid clinical application of CAR-T cells, the use of engineered T cells bearing a leukemia antigen-specific TCR gene directed against leukemia remains in its infancy. This may be due to the limitation of TCR transferred technique, etc. The difference between CAR-T and TCR-T cells was listed in Fig. 1. Recently, the first clinical trial using Wilms’ tumor gene product 1 (WT1)-TCR-modified T cells for leukemia immunotherapy has been reported [9, 10]. It has been said that CAR- and TCR-modified T cells (TCR-T cells) enter main street and Wall Street [6]. In this review, we summarize the current status of anti-leukemia immunotherapy using engineered T cells carrying leukemia antigen-specific TCR genes.

## Identification of leukemia-specific TCRs

To construct TCR-modified T cells, the isolation of TCRs that specifically recognize leukemia-specific antigen (LSA) or leukemia-associated antigen (LAA) epitopes is the first step. The TCRs could be identified in T cell clones possessing potent activity against leukemia cells either from the blood or bone marrow of leukemia patients [11] or from healthy donor T cells induced by LSA or LAA peptides with major histocompatibility complex (MHC) class-I/II-restriction. For example, WT1 is constitutively expressed in myeloid leukemia cells, including acute myelocytic leukemia (AML) and CML, and myelodysplastic syndrome (MDS), and WT1-specific CTLs have been identified in blood from leukemia patients [12, 13]. Thus, WT1 is an attractive target for inducing CTLs against leukemia for immunotherapy. Increasing numbers of leukemia-specific TCRs have been recently identified, including MHC class I/II-restricted TCRs with specificity for Formin-like protein 1 (FMNL1) with potent activity against CLL cells [20]. TERT is overexpressed in greater than 80% of primary tumors and leukemia cells. High-avidity TCRs specific for human TERT have been identified in AML, B-cell acute lymphoblastic leukemia (B-ALL), and adult T-cell leukemia (ATL) [4, 21, 22]. Additionally, aurora kinase A (AURKA)-specific TCRs [23, 24], murine double-minute 2 oncoprotein (MDM2)-TCRs [25, 26], and B cell-specific transcription factor BOB1-TCRs have been found in multiple myeloma [27], and hyaluronan-mediated motility receptor (HMMR/Rhamm)-TCRs were identified in acute lymphatic leukemia (ALL) and AML [28] (Table 1).

**Table 1:** Researches about leukemia-specific TCRs

Leukemia-specific TCRs	T cell type	HLA restriction	Leukemia	Research references
WT1-TCRs	CD4^+^ T cells CD8^+^ T cells	HLA-A2 HLA-DPB1*05:01 HLA-A*2402^+^	AML, CML, MDS	[14–19]
FMNL1-TCRs	CD4^+^ T cells	HLA-DRB1*0101 HLADRB1*1101	AML, lymphoma, myeloma	[20]
TERT-TCRs	CD34^+^ cells PBMC CD8^+^ T cells	HLA-A*0201 HLA-A*24:02 HLA-A2	B-CLL, ATL, AML, B-ALL	[4, 21, 22]
AURKA-TCRs	CD8^+^ T cells	HLA-A*0201	T-ALL, ATL	[23, 24]
MDM2-TCRs	CD4^+^ T cells CD8^+^ T cells	HLA-A*0201	AML, T lymphocytes	[25, 26]
BOB1-TCRs	PBMC CD4^+^ T cells CD8^+^ T cells	HLA-B*07:02	Lymphocytic B-cell, CLL, ALL, mantle cell lymphoma, MM	[27]
HMMR/Rhamn-TCRs	CD8^+^ T cells	CD8^+^ T cells	AML	[28]

*PBMC* peripheral blood mononuclear cell

## Anti-leukemia TCR-T cell construction

There are two important steps involved in developing TCR-T cell immunotherapy, obtaining the numbers of leukemia antigen-specific TCRs for TCR-T construction and establishing high-affinity tumor antigen-specific TCR gene modified T cells. In addition, it is necessary to provide a potential mechanism for overcoming the limitations of generating sufficient numbers of tumor antigen-specific T cells for each patient in vitro [8, 29]. A typical study involves generating replication-deficient retroviral vectors using the well-characterized OT-1 TCR genes and transducing murine T cells. Large numbers of antigen-specific T cells could be expanded and have been shown to be functionally active against tumor cells expressing the relevant antigen [30].

One of the important goals of T cell immunotherapy is establishing a persistent memory response to prevent disease relapse; however, the long-term function of TCR-T cells is limited due to reduced expression of introduced TCRs in quiescent resting T cells in vivo [31]. One solution to this issue is introducing TCRs with known endogenous specificity into T cells. Thus, stimulation through the endogenous TCR can increase the expression of the introduced TCR and subsequently activate the TCR-T cells. This method potentially provides a strategy for increasing the numbers of tumor-reactive T cells in a host and restoring more potent antitumor activity [31]. However, TCR gene transfer results in competition for surface expression and inappropriate pairing between exogenous and endogenous TCR chains, resulting in suboptimal activity and potentially harmful, unpredicted antigen specificities for the resultant TCRs. The endogenous TCRs compete with transgenic TCRs for surface expression and allow mixed dimer formation. Mixed dimers, formed by mispairing between endogenous and transgenic TCRs, may harbor autoreactive specificities. To avoid the possibility of transferred TCRs mispairing with endogenous TCRs, a key strategy is enhancing the expression of the transferred TCR and repressing the expression of the endogenous TCR α and β genes. Such TCR-edited T cells have been proven to be safer and more effective than that used in conventional TCR gene transfer: (1) generation of dominant TCR constructs that can suppress the expression of endogenous TCRs on the surface of transduced T cells [15]; (2) editing antigen-specific T cells by zinc finger nucleases (ZFNs) that promote disruption of the endogenous TCR β and α genes e.g., T cells treated with ZFNs lacked surface expression of CD3-TCRs, and after transferring a specific WT1-TCR, these TCR-edited T cells expressed WT1-TCR at high levels and did not mediate off-target reactivity but maintained their anti-WT1^+^ tumor activity in vivo [32]; (3) developing a novel and clinically feasible TCR “single editing” (SE) approach, which is based on disruption of only the endogenous TCR α chain followed by the transfer of genes encoding a tumor-specific TCR [33]; (4) a novel retroviral vector system encoding silencers (e.g., siRNAs) of endogenous TCR genes (siTCR vectors) e.g., WT1-siTCR gene-transduced T cells from leukemia patients successfully lysed autologous leukemia cells but not normal hematopoietic progenitor cells [34], and (5) using clustered, regularly interspaced short palindromic repeats-associated 9 (CRISPR/Cas9) technology to knockout endogenous TCRβ simultaneously with transduction of a cancer-reactive receptor of choice. TCR + CRISPR-modified T-cells were up to 1000-fold more sensitive to antigens than standard TCR-modified T cells or conventional model proxy systems used for studying TCR activity [35].

In general, TCR-T cells have mainly been constructed using the approach of transferring TCRα or β genes into αβT cells. However, to circumvent TCR mispairing, the development of TCR-modified T cells from other cell sources is a novel strategy: (1) TCRαβ-engineered γδT cells mediate effective anti-leukemic reactivity because γδ TCRs are not capable of forming dimers with αβ TCRs. Thus, transferring αβ TCRs into γδ T cells generate potent effector T cells for leukemia immunotherapy without expressing a potentially hazardous mix of TCR dimers [36]; (2) transduction of a pan-cancer reactive γδ TCR with CRISPR/Cas9 knockout of endogenous αβ TCRs in CD4^+^ and CD8^+^ T cells resulted in more efficient TCR-T cells against a panel of leukemia [35]; (3) introduction of TCRαβ genes into hematopoietic stem cells (HSCs) that could be further promoted to differentiate into specific T cells in vivo [37]; and (4) TCR-T cells derived from reprogrammed T cells [38] (Table 2).

**Table 2:** The strategies for avoiding transferred TCR mispairing with endogenous TCRs

Strategies	Research references
Editing TCRs	
Generating dominant TCR constructs that can inhibited the endogenous TCRs	[15]
Promoting the disruption of the endogenous TCRs	[32]
Disrupting only the endogenous TCR α chain	[33]
Silenced endogenous TCR genes	[34]
Knockout endogenous TCRβ simultaneously transduction of transferred TCRs	[35]
Using different kinds of cells	
γδT cells	[36]
CD4^+^ and CD8^+^ T cells without endogenous αβ TCRs	[35]
Hematopoietic stem cells	[37]
Reprogrammed T cells	[38]

## TCR-T cells for leukemia immunotherapy in preclinical studies

Increasing studies of TCR-T cells targeting different leukemia-relative antigens in different subtypes of leukemia have reported.

### Telomerase (TERT)-TCR-T cells

Human telomerase reverse transcriptase (hTERT) is a ribonucleoprotein enzyme, and its deregulation is a common step in leukemia; therefore, treatments targeting telomerase might be useful for leukemia therapy. Based on the finding that TERT-specific CTLs have been identified in the blood of B-CLL patients and TCRs with high avidity for human TERT could be isolated from TERT-vaccinated transgenic mice, adoptive HLA-A2-TERT-TCR-T cells could control B-CLL progression without severe side-effects in humanized mice. Moreover, TERT-TCR-T cells were also demonstrated to limit the progression of AML and B-ALL [4, 22]. Allogeneic or autologous CD8^+^ T cells modified by hTERT-siTCR in which HLA-A*24:02-restricted-hTERT_461–469_ peptide-specific TCRs were inserted into a novel retroviral TCR expression vector encoding small interfering RNAs directed against endogenous TCR genes in redirected T cells (hTERT-siTCR vector) were shown to successfully kill ATL cells without perturbing normal cells, including hematopoietic progenitors, in an HLA-A*24:02-restricted manner in vitro and in vivo [21]. Thus, the findings provide a new platform of TERT-based adoptive T cell therapy for leukemia with overexpression of TERT, particularly for patients who are unable to receive HSCT. Further investigation in clinical trial is expected, moreover, the effect of TERT-TCR-T cells on myeloid leukemia is needed to evaluate.

### AURKA-TCR-T cells

AURKA is overexpressed in leukemia cells including in ATL. AURKA-specific CTLs can specifically and selectively lyse leukemia cells. HLA-A*0201-restricted AURKA_(207–215)_-specific TCR-CD8^+^T cells have been demonstrated to lyse AURKA-overexpressing human HLA-A*0201 + leukemic cells, but they did not perturb normal HLA-A*0201 + cells, including hematopoietic progenitors. Furthermore, AURKA_(207–215)_-specific TCR-CD4^+^T cells demonstrated target-responsive Th1 cytokine production. AURKA_(207–215)_-specific TCR-CD8^+^ T cells also displayed anti-leukemia efficacy in a xenograft mouse model. Therefore, AURKA-TCR-T cell therapy against leukemia is an alternative approach [23, 24].

### HA-1-TCR-T cells

Minor histocompatibility antigen (HA)-specific graft-versus-leukemia (GVL) reactivity is observed following unselected donor lymphocyte infusion (DLI) for the treatment of leukemia relapse after allogeneic stem cell transplantation (allo-HSCT). Thus, HA-T cells are thought as a novel resource for developing T cell immunotherapy to manage post-HSCT leukemic relapse. HA-1-specific CD8^+^ CTLs were first identified in 2005 in unstimulated CD8^+^ T cells from healthy donors induced by artificial antigen-presenting cells (aAPCs) coated with anti-CD28 antibody (Ab) and HA-1 peptide, while the TCR repertoire of HA-1 tetramer-positive CTLs was identified as being oligoclonal with prominent usage of Vβ6 [39]. Subsequently, HLA-A2/HA-1-TCR gene transfer was used to generate HA-1-TCR-T cells from adult donor and cord blood T cells. The redirected T cells demonstrated hematopoietic-restricted cytolytic activity against HLA-A2^+^/HA-1^+^target cells, including leukemic cells, and may be exploited in immunotherapeutic settings of HSCT and cord blood transplantation (CBT) for hematologic malignancies [40]. Recently, the efficacy and safety of engineered HA-1-T cells has been established. These cells include a therapeutic transgene incorporating four components: an HA-1 specific TCR, a CD8 co-receptor to promote the function of class I-restricted TCRs in CD4^+^ T cells, an inducible caspase 9 safety switch (to enable the elimination of HA-1 TCR T cells in case of toxicity), and a CD34–CD20 epitope to facilitate the selection of the engineered cell products and tracking of transferred HA-1 TCR T cells. Moreover, the T cell products include HA-1 TCR CD4^+^ T cells, which are used to augment the persistence and function of HA-1 TCR CD8^+^ T cells and included only memory T cells [2]. Thus, HA-1 TCR-T cells are expected as special T cell immunotherapy to overcome post-HSCT leukemic relapse and to further evaluate in clinical trial.

### BOB-1, HMMR and MDM2-TCR-T cells

B cell-specific transcription factor BOB1-HLA-B*07:02-TCR-engineered T cells was efficiently shown to lyse primary B-cell leukemia, mantle cell lymphoma, and multiple myeloma in vitro and had in vivo antitumor reactivity in a multiple myeloma xenograft mouse model. This strategy may provide a novel target cellular treatment option for patients with B cell malignancies [41]. HMMR/Rhamm is overexpressed in numerous types of cancer, including ALL and AML. HMMR-specific, TCR-modified effector memory T cells could specifically recognize tumors and inhibit tumor outgrowth in a humanized xenograft mouse model and retard the outgrowth of disseminated AML. However, these HMMR-TCR-T cells demonstrated on-target killing of HLA-A2^+^ HSCs, indicating that the potential use of HMMR-TCR-T cell therapy is limited for MHC-mismatched HSC transplantation in which HLA-A2 differences can be used to restrict the recognition of patient HSCs and leukemia [28].

In addition, specificity for the human homolog of murine double-minute 2 (MDM2) oncoprotein by TCR-modified T cells was shown to be useful for broad-spectrum immunotherapy in malignant disease [25, 26].

## Anti-leukemia WT-1 TCR-T cells in clinical trials

WT1 is constitutively expressed in AML, CML, and MDS as well as in solid tumors such as breast cancer, and WT1^+^CTLs have been identified in peripheral blood of patients. Therefore, WT1 protein is an attractive target for immunotherapy, and a WT1 peptide vaccine was used for active immunotherapy in CML in a phase I trial [16, 17]. While WT1-specific CTLs and WT1-TCRs were identified many years ago [15], WT1-TCR gene-modified T cells eliminating leukemia cells were demonstrated in vitro and in vivo in a xenograft mouse model and leukemia-bearing NOD/SCID mice [14, 19]. There was also a study using the TCRα and β genes from high avidity CTLs specific for a WT1-derived peptide presented by HLA-A2 to modify T cells [14]. A number of studies have reported WT-1 special TCR genes transferred into CD8^+^ T cells or both CD4^+^ and CD8^+^ T cells. WT1-TCR-CD4^+^T cells display helper activity for WT-1-specific CTL induction and cytotoxicity against leukemia cells [19]. HLA-DPB1*05:01-WT1_332_-TCR-modified-CD4^+^ T cells displayed a strong proliferative response and Th1-type cytokine production in response to WT1_332_ peptide, WT1 protein, or WT1-expressing tumor cell lysate and lysed HLA-DPB1*05:01^+^ WT1^+^ human leukemia cells via the granzyme B/perforin pathway. Furthermore, WT1_332_-TCR-CD4^+^ T cells can enhance the induction of WT1_235_m-CTLs when stimulated with peripheral blood mononuclear cells with both HLA-A*24:02-CTL-epitope peptide (modified 9-mer WT1_235_ peptide, WT1_235_m) and WT1_332_ helper peptide. Thus, the feasibility of T cell therapy based on the adoptive infusion of WT1_332_-specific TCR-transduced CD4^+^ T cells could be employed for leukemia immunotherapy [18]. Moreover, WT1-siTCR/CD4^+^ T cells and WT1-siTCR/CD8^+^ T cells modified with a retroviral vector expressing HLA-A*24:02-restricted and WT1-specific TCR-α/β genes and siRNAs directed against endogenous TCRs have been developed. WT1-siTCR/CD4^+^ T cells sufficiently recognized leukemia cells in an HLA class I-restricted manner and provided target-specific Th1 help for WT1-siTCR/CD8^+^ T cells. In a xenograft mouse model, it was shown that WT1-siTCR/CD4^+^ T cells migrate to leukemia sites and subsequently attract WT1-siTCR/CD8^+^ T cells via chemotaxis. Importantly, WT1-siTCR/CD4^+^ T cells have been correlated with longer survival and enhanced formation of memory T cells. These results indicate the co-effects of TCR-modified CD4^+^ and CD8^+^T cells in anti-leukemia therapy [19].

Based on a wide range of investigation of WT1-TCR-T cells, recently, the first clinical trial using WT1-TCR-modified T cells for leukemia therapy was reported. Tawara et al. [10] reported a study on HLA-A*24:02-TCR-T cells infused in eight patients with refractory AML and high-risk MDS to evaluate safety and elucidate the kinetics of the T cells. During the study period, the TCR-T cells could be detected in blood for approximately 2 months, and no adverse events involving normal tissue were observed. The persisting TCR-T cells maintained ex vivo peptide-specific immune reactivity. Four of five patients who had persistent T cells at the end of the study survived more than 12 months. These results were the first to demonstrate the potential application of WT1-specific TCR-T cells in the clinic. As known, unlike B cell leukemia, it is limited target therapies for AML, undoubtedly, WT1-TCR-T cells are promising to treat refractory and relapse AML patients, particularly for the older AML patients who are unable to receive HSCT.

## Summary and future directions

The transfer of TCR genes into primary human T cells to endow their specificity toward leukemia cells is becoming an interesting tool for T cell immunotherapy for hematological malignancies. However, there is still much to be considered. Although the development of TCR-T cells began as early as the 90 s, their application falls behind that of CAR-T cells, which was thriving at the end of 20 century. The advantage of TCR-T cells may be the high level of avidity and efficacy of TCRs, which can be a valuable addition to current treatment options for patients suffering from insufficient extracellular low leukemia antigens [42]. The lower incidence of cytokine release syndrome may be another advantage of TCR-T cells as well. However, some challenges might include the following: (1) preventing mis-pairing between introduced and endogenous TCRs, causing off-target reactivity, (2) the choice of T cell subpopulations for gene transfer, (3) overcoming exhausted and senescent T cells in patients, enhancing transgenic T cells responding to antigens, and (4) the promotion of persisting gene-modified T cells in vivo [43–45]. After overcoming the limitation, TCR-T cells may have bright future for leukemia immunotherapy.

## Abbreviations

HSCT: hematopoietic stem cell transplantation
CTL: cytotoxic T lymphocyte
CML: chronic myelogenous leukemia
CMV: cytomegalovirus
EBV: Epstein–Barr virus
PTLPD: post-transplantation lymphoproliferative disorder
TERT: telomerase
B-CLL: B-cell chronic lymphocytic leukemia
CAR: chimeric antigen receptor
TCR: T cell receptor
CD19: cluster of differentiation 19
HLA: human lymphocyte antigen
MART-1: melanoma-associated antigen peptide-1
WT1: Wilms’ tumor gene product 1
LSA: leukemia-specific antigen
LAA: leukemia-associated antigen
MHC: major histocompatibility complex
AML: acute myeloid leukemia
MDS: myelodysplastic syndrome
FMNL1: Formin-like protein 1
B-ALL: B-cell acute lymphoblastic leukemia
ATL: adult T-cell leukemia
AURKA: aurora kinase A
MDM2: murine double-minute 2 oncoprotein
HMMR/Rhamm: hyaluronan-mediated motility receptor
ALL: acute lymphatic leukemia
ZFNs: zinc finger nucleases
SE: single editing
CRISPR/Cas9: clustered regularly interspaced short palindromic repeats-associated 9
HSCs: hematopoietic stem cells
hTERT: human telomerase reverse transcriptase
HA: histocompatibility antigen
GVL: graft-versus-leukemia
DLI: donor lymphocyte infusion
allo-HSCT: allogeneic hematopoietic stem cell transplantation
aAPCs: artificial antigen-presenting cells
Ab: antibody
CBT: cord blood transplantation
MDM2: murine double-minute 2
